# Family physicians’ knowledge, beliefs, and practices in promoting healthy lifestyles and weight management for obese patients in Tabuk, Saudi Arabia

**DOI:** 10.3389/fendo.2024.1456086

**Published:** 2024-11-22

**Authors:** Amirah M. Alatawi, Mansuor A. Alanazi, Maram Ati Almohammadi

**Affiliations:** ^1^ Department of Family and Community Medicine, Faculty of Medicine, University of Tabuk, Tabuk, Saudi Arabia; ^2^ Training Department, Academic Affairs and Training Administration, Ministry of Health, Tabuk, Saudi Arabia

**Keywords:** obesity, knowledge, beliefs, practice, family physicians

## Abstract

**Background:**

Obesity is one of the most prevalent and relevant health problems in Saudi Arabia and requires urgent attention. Family physicians are the first point of contact and one of the most important starting points for the successful treatment of being overweight or obese.

**Aim:**

This study aimed to assess the knowledge, beliefs, and practices of primary care physicians in promoting healthy lifestyles and physical activity among obese patients.

**Methods:**

This cross-sectional study included family physicians who were board-certified or registered in a family medicine training program and working at government family healthcare centers in Tabuk. A predesigned structured questionnaire was distributed either in a printed form or as an online survey.

**Results:**

The study included 83 family physicians. Of these, 84.1% agreed that overweight and obese patients should be screened for dyslipidemia, and 67.5% agreed to offer advice on weight control even if the patient did not ask for it, demonstrating what family medicine physicians thought of physical inactivity (97.6%). Assessment of the obesity counseling practices of family medicine physicians revealed that approximately three-fourths (75.9%) always calculated the body mass index (BMI) of patients during obesity management, while comprehensive counseling regarding physical activity was higher (81.9%). The system for providing educational materials as part of managing overweight or obesity was found to be inadequate as 25.3% had never done so. The family medicine physicians had sufficient knowledge and positive beliefs about obesity management but also improper counseling practices. A lack of training and poor patient compliance with management plans are the main barriers to controlling obesity levels.

## Introduction

Obesity is the primary cause of cardiovascular diseases (CVD), diabetes mellitus (DM), cancers, and ischemic heart disease (IHD); therefore, it is considered a major non-communicable disease in Saudi Arabia, with a high prevalence and great financial burden that is beyond the capacity of the people and government to cope with (1). Obesity is defined as abnormal or excessive fat accumulation that may impair health (2). Body mass index (BMI) is a simple index of weight and height that is commonly used to classify being overweight and obesity in adults.

Although obesity is preventable, globally, the prevalence of obesity among adults ranges from 42.4% in the United States of America to 59% in Europe, and one-third of children are obese (2).

A review of obesity prevalence in Saudi Arabia by Al-Qarni classified Saudi Arabia as having the highest prevalence in the Gulf region, with approximately one in four people being obese in 2020 (1, 3). A patient who is obese or overweight needs an effective counseling intervention, hence, family physicians are expected to provide conscious counseling in terms of individual prescriptions of optimal dietary items, calories, and physical exercise per day according to the country’s guidelines. This cross-sectional study assessed family physicians’ beliefs, knowledge, and practices in a quantitative manner. Furthermore, we explored their opinions, experiences, and beliefs regarding counseling for obesity and being overweight at primary healthcare centers (PHCs) in the Tabuk region. Due to the scarcity of data on physical activity counseling practices in PHCs in Saudi Arabia (4), we focused on the assessment of physical activity knowledge and attitudes of health providers at the PHC level; hence, we could correctly identify and address barriers to physical activity counseling.

Moreover, physical activity counseling at the PHC level is internationally recognized as a valuable and cost-effective approach to combat obesity and its associated comorbidities. According to Saudi guidelines for the management of obesity in adults in 2016, a well-organized and practical program for lifestyle intervention at the primary healthcare level is highly recommended. Likewise, the guidelines suggest individual counseling rather than providing generic educational materials and physical activity rather than no activity; thus, it encourages counseling for physical activity with diet rather than diet alone (5). In contrast, a systematic review of childhood obesity conducted by (6) classified clinic-based lifestyle interventions for obesity and revealed favorable results, suggesting family involvement and a multidisciplinary approach to be advantageous. Furthermore, student counseling is a valuable intervention option to combat obesity among school-age children in the long term (7). The study by Ghoraba et al. (8) forecasted a rising prevalence of diabetes in the coming years owing to the high prevalence of prediabetes in Saudi Arabia (23.6%); thus, the study proposed lifestyle interventions to reduce new incident cases.

This study aimed to assess the knowledge, beliefs, and practices of family physicians in promoting healthy dietary habits and physical activity among patients with obesity in Tabuk, Saudi Arabia.

## Methods

### Ethical considerations

This study was approved by the Ethics Committee of the University of Tabuk in Tabuk, Saudi Arabia (RB Protocol No: TU-07710221164). Informed consent was obtained from all the participants, including a consent statement from the survey. The participants were clearly informed about the nature and objectives of the study, and were also notified that their anonymized data would be used only for research purposes and that their confidentiality would be maintained.

### Study design, setting, and date

This cross-sectional survey was conducted at 25 government primary healthcare centers in Tabuk City, Saudi Arabia, between May and June 2022.

### Sample size and sampling technique

**Figure 1:**
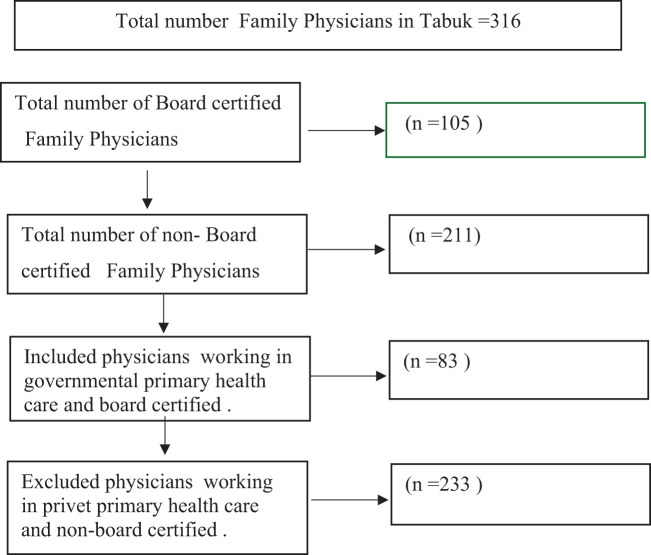
Flow chart of the inclusion and exclusion criteria.

The sample size was calculated using the following formula: n=Z2 P Q/d2, where Z = 95% confidence (1.96) and P = number of family physicians and residents in Tabuk City, with a 5% confidence interval. The sample size was calculated to be 83 participants (**Figure 1**).

The unit of analysis was the family medicine physician, and the sampling followed the rule of dual-frame sampling for a standalone facility survey approach (WHO, 2003). Therefore, the sampling of the healthcare centers followed a simple random sampling technique. An updated list of healthcare facilities was used to create a list frame sample for facilities and an area frame sample was used to balance coverage through the allocation of healthcare facilities, using an updated map from the Ministry of Health. All 25 government primary healthcare centers in Tabuk City were included in the study.

### Eligibility criteria

Family physicians who were board-certified or registered in the family medicine training program and working at government family healthcare centers in Tabuk were included in the study. Family physicians who were not board-certified or registered in the family medicine training program and those working at private family healthcare centers were excluded.

### Data collection tool

A pre-designed structured questionnaire was used to collect data. The questionnaire was validated using two previously published research articles, with some minor modifications (9–11). The questionnaire consisted of 37 items. Part 1 consisted of seven statements regarding personal data, including age, gender, nationality, experience, average number of patients per day, and final qualifications. Part 2 contained nine statements that tested knowledge of the health risks of obesity, diagnosis, and lifestyle (diet and exercise). Responses to the statements were categorized as “Yes” for affirmative answers, “No” for non-affirmative answers, and “Not sure” at the GP’s discretion. Part 3 used a 10-point scale to measure responses, with an attitude rating on a five-point Likert scale (strongly agree, agree, neither, disagree, and strongly disagree) and consisted of statements. Item agreement scores were calculated using a scale [minimum of one (completely disagree) to a maximum of five (completely agree)]. Agreement was scored as 4 or 5 (agree and strongly agree), disagreement was scored as 1 or 2 (disagree and strongly disagree), and neutrality was scored as 3. Part 4 consisted of 10 statements to measure responses related to barriers to the adequate management of overweight and obesity with answers of “Agree”, “Neither”, or “Disagree.” Part 5 consisted of nine statements that measured responses to (always, often, or never) assessing practice. We also asked about possible causes of obesity using a list of 10 possible causes of obesity.

### Procedure

The questionnaires were distributed in either a printed or an online form. The participants were met at their workplace by the co-authors, and all family physicians, including residents, registrars, and consultants, were invited to participate after a general explanation of the study’s aim.

### Statistical analysis

The collected data were recorded, verified, and tabulated using the Statistical Package for Social Sciences (IBM SPSS Statistics), version 22.0 for Windows (IBM Corp., Armonk, N.Y., USA). Descriptive statistics (frequencies and percentages) were calculated. Inferential statistics were calculated using the chi-square test for the associations between age groups, gender, qualifications, and the practices of the respondents. Differences were considered statistically significant at P < 0.05.

## Results

This study included 83 family medical physicians. Women constituted 63.9% of the total population. The most common age group was <35 years (56.6%). Saudi physicians accounted for 56.6% of the sample, whereas non-Saudi physicians accounted for 43.4%. Most participants were residents (57.8%), senior registrars (18.1%), consultants (18.1%), or registrars (6.0%). Most had clinical experience of 1-5 or 6-10 years (45.8% and 26.5%, respectively), followed by 11-15 years (18.1%). Finally, 30 (36.1%) and 29 (34.9%) physicians observed fewer than 50 and 50-100 patients per week, respectively (**Table 1**).

**Table 1:** Sociodemographic and professional characteristics of the study participants.

		N= 83	%
Age, years	<35	47	56.6%
	36-40	18	21.7%
	41-45	10	12.0%
	>45	8	9.6%
Gender	Women	53	63.9%
	Men	30	36.1%
Nationality	Saudi	47	56.6%
	Non-Saudi	36	43.4%
Qualification	Family medical resident	48	57.8%
	Senior registrar	15	18.1%
	Consultant	15	18.1%
	Registrar	5	6.0%
Experience in family medicine practice (years)	1-5	38	45.8%
	6-10	22	26.5%
	11-15	15	18.1%
	>15	8	9.6%
The average number of patients seen per week	<50	30	36.1%
	51-100	29	34.9%
	>100	24	28.9%

**Table 2** shows the knowledge of family medicine physicians regarding being overweight or obese and the necessity of weight loss. The majority (96.3%) knew that obesity was defined as a BMI > 30 kg/m^2^, and all (100%) physicians knew that patients with type 2 diabetes should be evaluated for being overweight or obese. Furthermore, 92.7% realized that 10% weight loss was effective in preventing the progression of prediabetes to type 2 diabetes, whereas 81.7% understood that 5%–15% weight loss was effective in treating polycystic ovary syndrome. There was insufficient knowledge about the value of weight loss in decreasing hepatic inflammation, hepatocellular injury, and fibrosis by 10–40%; 46.9% knew this, while 43.2% did not. In addition, 17.3% of the participants did not know that >10% weight loss was effective in treating infertility in women with obesity. Additionally, 77 respondents (93.9%) knew that counseling overweight or obese clients should include investigating risk factors for cardiovascular disease, while 3.7% did not know. Most of the participants (96.3%) recognized the benefit of measuring blood pressure in all overweight or obese patients by screening for the presence of hypertension or prehypertension, whereas 84.1% agreed that overweight and obese clients should be screened for dyslipidemia.

**Table 2:** Knowledge of family medicine physicians about obesity.

		N	%
Obesity is defined as a BMI exceeding 30 kg/m2.	No	3	3.7%
	Yes	78	96.3%
Patients with T2DM should be evaluated for the presence of overweight or obesity.	Yes	82	100.0%
Risk factors for cardiovascular disease should be assessed in patients with overweight or obesity.	I do not know	3	3.7%
	No	2	2.4%
	Yes	77	93.9%
Weight loss of 10% is effective in treating diabetes risk (i.e., prediabetes, metabolic syndrome) and preventing progression to type 2 diabetes.	I do not know	5	6.1%
	No	1	1.2%
	Yes	76	92.7%
All overweight or obese patients and individuals experiencing progressive weight gain should be screened for dyslipidemias.	I do not know	2	2.4%
	No	11	13.4%
	Yes	69	84.1%
Blood pressure should be measured in all overweight or obese patients to screen for the presence of hypertension or prehypertension.	I do not know	1	1.2%
	No	2	2.4%
	Yes	79	96.3%
Weight loss of 5% to 15% is effective in treating polycystic ovary syndrome (PCOS).	I do not know	11	13.4%
	No	4	4.9%
	Yes	67	81.7%
Weight loss as high as 10 to 40% may be required to decrease hepatic inflammation, hepatocellular injury, and fibrosis.	I do not know	35	43.2%
	No	8	9.9%
	Yes	38	46.9%
Weight loss of >10% is effective in treating infertility in women with obesity.	I do not know	14	17.3%
	No	1	1.2%
	Yes	66	81.5%

The beliefs of the family medicine physicians regarding the management of obesity and being overweight are presented in **Table 3**. In total, 59 (71.1%) physicians agreed with the chronic nature of obesity, whereas a high percentage (98.8%) agreed with the necessity of health education for obese patients regarding the health risks of obesity. However, only 67.5% agreed to offer advice on weight control even if the patient did not ask for it. Among the study participants, 61.4% felt competent in prescribing weight loss programs for obese patients, less than half (48.2%) agreed that they were usually successful in helping obese patients lose weight, 42.2% were neutral, and 79.5% agreed to recommend an evaluation by a surgeon if a patient met the appropriate criteria for obesity surgery. However, 38.6% thought that the role of family medicine physicians was to refer overweight or obese patients to other professionals rather than to attempt to treat them, and 51.8% felt that primary healthcare centers were not well prepared to manage overweight or obese patients. Furthermore, 64.1% believed that the long-term maintenance of weight loss was impossible in most obese patients.

**Table 3:** Beliefs of family medicine physicians about obesity management.

		N= 83	%
I believe it is necessary to educate obese patients about the health risks of obesity.	Agree	82	98.8%
	Neutral	1	1.2%
Obesity is a chronic disease.	Agree	59	71.1%
	Disagree	15	18.1%
	Neutral	9	10.8%
I will only offer advice about weight control if the patient asks for it.	Agree	18	21.7%
	Disagree	56	67.5%
	Neutral	9	10.8%
I feel competent in prescribing weight loss programs for obese patients.	Agree	51	61.4%
	Disagree	9	10.8%
	Neutral	23	27.7%
Most obese patients are well aware of the health risks of obesity.	Agree	17	20.5%
	Disagree	40	48.2%
	Neutral	26	31.3%
If a patient meets the appropriate criteria for obesity surgery, I recommend an evaluation by a surgeon.	Agree	66	79.5%
	Disagree	9	10.8%
	Neutral	8	9.6%
I am usually successful in helping obese patients lose weight.	Agree	40	48.2%
	Disagree	8	9.6%
	Neutral	35	42.2%
For most obese patients, long-term maintenance of weight loss is impossible.	Agree	24	28.9%
	Disagree	29	34.9%
	Neutral	30	36.1%
I feel that primary health care centers are well prepared to manage overweight and obesity.	Agree	20	24.1%
	Disagree	43	51.8%
	Neutral	20	24.1%
The role of family medicine physicians is to refer overweight and obese patients to other professionals rather than attempt to treat them.	Agree	14	16.9%
	Disagree	51	61.4%
	Neutral	18	21.7%

The data displayed in **Table 4** demonstrates that family medicine physicians believed that physical inactivity (97.6%), endocrine disorders (84.3%), genetic factors (81.9%), overeating (80.7%), and a high-fat diet (75.9%) were the most frequent risk factors for obesity in Saudi Arabia. Moreover, a lack of training (72.3%) and poor patient adherence to the management plan (71.1%) were considered crucial barriers to obesity management in Saudi Arabia.

**Table 4:** Attitudes of family medicine physicians toward risk factors of obesity and barriers to obesity management in Saudi Arabia.

	N= 83	%
Physical inactivity	81	97.6%
Overeating	67	80.7%
High-fat diet	63	75.9%
Genetic factors	68	81.9%
Poor nutritional knowledge	58	69.9%
Psychological problems	62	74.7%
Repeated dieting (weight cycling)	26	31.3%
Restaurant eating	48	57.8%
Lack of willpower	15	18.1%
Metabolic defect	59	71.1%
Endocrine disorder	70	84.3%
Lack of training	60	72.3%
Lack of administrative support	38	45.8%
Poor healthcare system	35	42.2%
The high failure rate in reducing weight	45	54.2%
Lack of facilities	22	26.5%
Lack of a dietician	44	53.0%
Time constraints	20	24.1%
Poor patient adherence to the management plan	59	71.1%
Lack of physicians’ confidence in managing obesity	33	39.8%

The assessment of obesity counseling practices by family medicine physicians revealed that approximately three-fourths (75.9%) always calculated BMI during obesity management while measuring waist circumference was always performed by 14.5% and often performed by 51.8%. Check-up/treatment of comorbidities was always considered by 78.3% of physicians and was often performed by 20.5%. Comprehensive counseling practices for behavioral change were always provided by 67.5% and were often provided by 31.3%. Comprehensive counseling on diet was either always (66.3%) or often (33.7%) provided, although comprehensive counseling on physical activity was more common (81.9%). The system for providing educational materials as part of managing overweight or obesity was found to be inadequate as 25.3% had never done so. In obesity management, improper practices, such as a high percentage of referrals to dieticians (95.2%) and non-referral of obese patients for surgery when indicated (13.3%), were recorded (**Table 5**).

**Table 5:** Assessment of obesity counseling practices by the family medicine physicians.

		N= 83	%	P1	P2	P3
Assessment of BMI	Always	63	75.9%	0.872	0.133	0.022*
	Never	2	2.4%			
	Often	18	21.7%			
Waist circumference measurement	Always	12	14.5%	0.055	1.00	0.275
	Never	28	33.7%			
	Often	43	51.8%			
Check-up/treatment of comorbidities	Always	65	78.3%	0.691	0.531	0.921
	Never	1	1.2%			
	Often	17	20.5%			
Comprehensive counseling for behavior change	Always	56	67.5%	0.616	0.254	0.988
	Never	1	1.2%			
	Often	26	31.3%			
Comprehensive counseling on diet	Always	55	66.3%	0.256	0.029*	0.023*
	Often	28	33.7%			
Comprehensive counseling on physical activity	Always	68	81.9%	0.911	0.146	0.530
	Often	15	18.1%			
Refer your obese patients to dietitians for obesity management	Always	33	39.8%	0.077	0.122	0.692
	Never	4	4.8%			
	Often	46	55.4%			
Refer your obese patients for surgery if indicated	Always	41	49.4%	0.481	0.902	0.533
	Never	11	13.3%			
	Often	31	37.3%			
Provide educational materials as part of managing being overweight or obese	Always	27	32.5%	0.022*	0.804	0.929
	Never	21	25.3%			
	Often	35	42.2%			

*Significant at p<0.05.

P1: Association with age groups.

P2: Association with gender.

P3: Association with qualifications.

Inferential statistics revealed that the assessment of BMI was significantly higher among residents than among registrars and consultants (p=0.022). The practice of comprehensive counseling on diet was significantly higher among residents (p=0.023) and among women (p=0.029). Moreover, providing educational materials as part of managing being overweight or obese was significantly higher among physicians aged less than 35 years than among the older age groups (p=0.022) (**Table 5**).

## Discussion

Obesity is a major public health concern worldwide. Family medicine physicians are the first point of contact for patients and should have sufficient knowledge, attitudes, and beliefs about obesity control (12, 13). This study aimed to assess the practices, knowledge, and beliefs of family physicians regarding the promotion of healthy dietary habits and physical activity among obese or overweight patients in Saudi Arabia.

Our main findings demonstrate that the physicians had positive beliefs regarding obesity management. However, a lack of physician training and poor patient adherence to the management plan were the common barriers to obesity management. Insufficient counseling practices by the family medicine physicians were also noted. Young physicians provided inadequate educational materials for managing obesity.

Female Saudi residents aged less than 35 years with clinical experience of 1-5 or 6-10 years usually examined less than 50 and 50-100 patients per week, respectively. Comparable findings were reported by Alsaati and Almasaodi (14), who evaluated the knowledge, attitudes, and skills of family residents in Makkah regarding obesity. Sebiany (10) included 130 participants, with a mean age of 41 years and an average work experience of 12 years. Young physicians are suitable subjects for training and educational efforts that are intended to improve obesity management (15).

Most of the physicians had sufficient knowledge of the definition of obesity and subsequent obesity-related morbidities, including type 2 diabetes mellitus, cardiovascular diseases, dyslipidemia, polycystic ovary disease, and infertility. However, there was insufficient knowledge regarding the value of weight loss in reducing hepatic inflammation, hepatocellular injury, and fibrosis. Family medicine residents in Makkah were found to have sufficient knowledge of obesity management and the associated comorbidities (14). This could be attributed to the fact that the resident physicians had more recent degrees, updated their knowledge regularly, and had sufficient experience. Furthermore, sufficient knowledge about weight loss may be required to decrease hepatic diseases and could be improved by increasing physicians’ awareness and training regarding the benefits of obesity management (16).

In our study, most of the family medicine physicians believed that obesity is a chronic disease. Furthermore, most physicians had positive beliefs about obesity. They agreed to the necessity of health education for obese patients about the health risks of obesity through the offering of advice about weight control even if the patient does not ask for it, and prescribing weight loss programs. Primary care physicians are the primary point of contact for patients and should identify an obesity case, initiate treatment with lifestyle modifications and obesity drugs, and refer the patient for bariatric surgery when indicated (17). In the present study, the obesity knowledge of the physicians was fair. However, there is still room for improvement, in particular, regarding knowledge of the role of weight loss in the prevention of metabolic-associated fatty liver disease and polycystic ovary syndrome, and in dyslipidemia screening. In addition, nearly one-third of the family physicians did not view obesity as a chronic disease, 32.5% advised weight control only when asked by the patients, and 38.5% felt incompetent in prescribing weight loss programs to obese patients. Counseling regarding obesity treatment, the inclusion of lifestyle interventions and other effective obesity drugs such as glucagon-like receptor-1 agonists, and referring patients for bariatric surgery are vital and should be enforced by including them in the medical curriculum and through continuous education. Primary care physicians need to adhere to the guidelines for obesity management using a multidisciplinary approach. Importantly, Asian people are at higher risk of the development cardiovascular disease at a lower body mass index compared to the Western population (18, 19).

Several Saudi studies have reported similar findings to ours (14, 20, 21). In Norway (22), most general practitioners said that it was their responsibility to manage adults with obesity, and felt secure doing so. However, a Swedish study (23) showed a paradoxical attitude toward obesity among physicians. Although Swedish physicians were able to help obese individuals, most agreed that it was the patient’s obligation to lose weight. Another Swedish study (24) stated that physicians felt frustrated when trying to help obese individuals and referred them to undergo bariatric surgery. These discrepancies in physicians’ beliefs and attitudes can be attributed to the differences in populations and cultures.

In this study, the family medicine physicians believed that physical inactivity, endocrine disorders, genetic factors, overeating, and a high-fat diet are the most frequent risk factors for obesity. These factors can alter the ability of the hypothalamus to regulate one’s appetite, leading to increased hunger or delayed satiety (25). Similarly, Sebiany (10) and Khalifa et al. (26) reported that Saudi physicians are aware of the risk factors for obesity. The main constraints on obesity management in our study were related to the healthcare system and patient factors. In addition, a lack of training and poor patient compliance with management plans were the most common barriers reported in several studies (13, 19, 20, 25). Patient non-compliance could be explained by cultural, social, or psychological factors. Swedish primary care physicians stated that a lack of time and resources during doctor-patient consultations was the biggest barrier to discussing weight loss management with their patients (23). Meanwhile, an American study (27) revealed that the biggest obstacles to treating obesity were a lack of physician confidence and a lack of patient drive. These different barriers may be related to the different populations studied.

Assessment of BMI was significantly associated with qualification level. Registrants and consultants may be involved in other tasks, such as research or education of medical students, and may be up-to-date on recent guidelines. Residents may have insufficient time to check recent guidelines (28). A BMI assessment is recommended for the diagnosis and evaluation of obesity (29). Improper practices were reported regarding waist circumference measurement and non-referral of obese patients to dietitians or surgery when indicated. In Makkah, most physicians did not diagnose a patient as being overweight or obese based on waist circumference (14). Our study supported the previous findings because only a minority of the primary care physician measured the waist circumference of their patients, putting them at high risk of cardiovascular disease and mortality (30). The lower rate of waist circumference measurement is in line with a previous study. Waist circumference is a simple measurement that predicts cardiovascular disease and diabetes mellitus development and the inclusion of it in routine obesity management is encouraged (31).

Moreover, comprehensive counseling on diet was significantly higher among female physicians and was correlated with qualification level. Similarly, Foster et al. (32) reported that women had suitable attitudes and practices toward obesity management compared to men. Ferrante et al. (33) found no gender differences in physicians’ attitudes toward obesity management. In our study, young physicians provided inadequate educational materials for managing being overweight or obese. The finding that 25.3% of the physicians had never provided educational materials as part of obesity management is concerning as educational material is a promising intervention in obesity prevention (34). Plausible explanations could be a lack of available materials, insufficient training, or time constraints. Thus, training courses and providing educational materials are highly recommended. Furthermore, this could be explained by a lack of training and knowledge regarding recent guidelines. In contrast to our findings, Carrasco et al. (23) reported that young Swedish physicians had more recent training and were more knowledgeable about guidelines.

Our findings suggest that young family medicine physicians should receive training and education on obesity-related concerns to address obesity more successfully. Moreover, family medicine residents act as a bridge between different services for managing obesity and helping physicians analyze patients’ weight and health in relation to their weight status (35). Creating interdisciplinary teams for managing obesity and connecting family medicine residents to a wider array of other healthcare experts and programs would be helpful in obesity management.

### Limitations

The subjectivity of the self-administered questionnaire and the possibility that family medicine physicians discussed the findings among themselves represent the limitations of the current study. The sample is limited to family physicians in Tabuk, which may not reflect practices in other regions of the country. Variations in healthcare access, resources, and patient demographics across different regions could significantly impact the generalizability of the findings. Furthermore, larger, multicenter studies combining surveys with interviews or observational data are required. Improving the infrastructure within which family physicians operate and more comprehensive obesity management programs and patient follow-up systems are highly recommended.

## Conclusion

The family medicine physicians in Tabuk City have adequate knowledge about obesity and positive attitudes toward obesity management. However, their counseling practices were poor. The main obstacles to controlling obesity are a lack of training and poor patient adherence to the management plan. Training courses and the inclusion of obesity management in medical training is highly recommended.

## Data Availability

The original contributions presented in the study are included in the article/supplementary material. Further inquiries can be directed to the corresponding author.
